# Large and persistent subnational inequalities in reproductive, maternal, newborn and child health intervention coverage in sub-Saharan Africa

**DOI:** 10.1136/bmjgh-2019-002232

**Published:** 2020-01-26

**Authors:** Cheikh Mbacké Faye, Fernando C Wehrmeister, Dessalegn Y Melesse, Martin Kavao Kavao Mutua, Abdoulaye Maïga, Chelsea Maria Taylor, Agbessi Amouzou, Safia S Jiwani, Inácio Crochemore Mohnsam da Silva, Estelle Monique Sidze, Tyler Andrew Porth, Tome Ca, Leonardo Zanini Ferreira, Kathleen L Strong, Richard Kumapley, Liliana Carvajal-Aguirre, Ahmad Reza Hosseinpoor, Aluisio J D Barros, Ties Boerma

**Affiliations:** 1 West Africa Regional Office, African Population and Health Research Center, Dakar, Senegal; 2 Post-Graduate Programme in Epidemiology, Universidade Federal de Pelotas, Pelotas, Brazil; 3 Centre for Global Public Health, Department of Community Health Sciences, Countdown 2030 for Women’s, Children’s and Adoelscents’ Health, Winnipeg, Manitoba, Canada; 4 African Population and Health Research Center, Nairobi, Kenya; 5 International Health, Johns Hopkins University Bloomberg School of Public Health, Baltimore, Maryland, USA; 6 Department of Maternal, Newborn, Child and Adolescent Health, World Health Organization, Geneva, Switzerland; 7 International Center for Equity in Health, Universidade Federal de Pelotas, Pelotas, Brazil; 8 UNICEF, New York City, New York, USA; 9 West African Health Organisation, Bobo-Dioulasso, Hauts-Bassins, Burkina Faso; 10 Data and Analytics Section, UNICEF, New York City, New York, USA; 11 Division of Data, Analytics and Delivery for Impact, World Health Organization, Geneva, Switzerland

**Keywords:** maternal health, child health

## Abstract

Subnational inequalities have received limited attention in the monitoring of progress towards national and global health targets during the past two decades. Yet, such data are often a critical basis for health planning and monitoring in countries, in support of efforts to reach all with essential interventions. Household surveys provide a rich basis for interventions coverage indicators on reproductive, maternal, newborn and child health (RMNCH) at the country first administrative level (regions or provinces). In this paper, we show the large subnational inequalities that exist in RMNCH coverage within 39 countries in sub-Saharan Africa, using a composite coverage index which has been used extensively by Countdown to 2030 for Women’s, Children’s and Adolescent’s Health. The analyses show the wide range of subnational inequality patterns such as low overall national coverage with very large top inequality involving the capital city, intermediate national coverage with bottom inequality in disadvantaged regions, and high coverage in all regions with little inequality. Even though nearly half of the 34 countries with surveys around 2004 and again around 2015 appear to have been successful in reducing subnational inequalities in RMNCH coverage, the general picture shows persistence of large inequalities between subnational units within many countries. Poor governance and conflict settings were identified as potential contributing factors. Major efforts to reduce within-country inequalities are required to reach all women and children with essential interventions.

**Summary box**

- Despite national progress during the Millennium Development Goals era, large subnational inequalities in reproductive, maternal, newborn and child health (RMNCH) interventions coverage have persisted within most countries in sub-Saharan Africa.
- A wide range of inequality levels and patterns in RMNCH coverage exist. Several countries have low national coverage with large inequalities between subnational units, often with capital city top inequality, while others have high coverage with little inequality or intermediate coverage but with some regions that are left far behind.
- The study of inequality patterns provides critical guidance to ensure that essential interventions for RMNCH reach women and children equally in all subnational units.

## Introduction

During the Millennium Development Goals (MDGs) era (2000–2015), most countries in sub-Saharan Africa (SSA) made significant progress towards achieving reductions in maternal and child mortality. For instance, the maternal mortality ratio in SSA declined by 45% since 1990 and the annual rate of reduction of under-5 mortality 2000–2015 was 4.1%, two and half times faster compared with the preceding decade.^1 2^ While the MDGs focused on national progress, international efforts such as the Demographic and Health Surveys (DHS), Multiple Indicator Cluster Surveys (MICS) and the Countdown to 2015 for Reproductive, Maternal, Newborn and Child Health (RMNCH) showed the existence of major inequalities within countries by socioeconomic status and place of residence.^3–5^ Despite the limited attention to such in-country inequalities, the gaps in the coverage of RMNCH interventions declined considerably between rural and urban women and children, and between the poorest and the richest.^6^

The Sustainable Development Goals 2016–2030 provide a framework with greater attention for inequalities, aiming to reach all populations in need with quality essential health services. Progress towards universal health coverage requires evidence on who is left behind to enable health programmes distribute resources efficiently and effectively. Subnational disparities in the coverage of RMNCH interventions by administrative areas are one of the most critical and actionable dimensions of inequality.

Country administrative divisions, such as states, provinces, regions, districts or counties, are the main planning and implementation administrative units for service delivery by government and development partners. Several countries made special efforts to decentralise health systems.^7^ Solid evidence on subnational health inequalities is needed to inform policy-making, allocate resources, target programmes and ensure accountability.

Data to generate such evidence primarily come from routine health information systems and national surveys. Routine health facility data can be an important source of information to track intervention coverage trends. However, there are major challenges due to data quality issues, particularly at subnational level.^8^ Coverage statistics based on health facility data are further compounded by uncertainties about the size of the target population such as the number of live births or children eligible for immunisation.

Generally, surveys produce standardised high-quality coverage and other population statistics that can be compared over time and between countries. In this paper, we focus on the evidence from household surveys, notably DHS and MICS.^4 5^ We reviewed the literature and synthesised evidence on subnational inequalities for 39 countries in SSA. The DHS and MICS use sample designs that aim to generate estimates at administrative level 1 (mostly referred to as province or region) for key health indicators. Some surveys have adequate sample sizes to compute coverage statistics at administrative level 2 (such as district or county), such as the Malawi DHS 2016 and Kenya DHS 2014. In this paper, we focus on administrative level 1.

## Use of evidence on subnational inequalities

Country planning and monitoring documents often pay attention to subnational inequalities in health indicators, based on health facility data and surveys. Our review of national health sector strategic plans and the associated monitoring and evaluation plans and practices in 10 countries showed that countries often have targets to reduce subnational inequalities, but systematic monitoring of progress is limited.^9^ Immunisation programmes are an exception where district-level coverage monitoring is often a core element.^10^

Research studies may use data on subnational variation in coverage, either as a determinant of progress in child mortality or nutrition, or as an indicator of system performance itself.^11–13^ Most studies, however, focus on socioeconomic and urban–rural inequalities in RMNCH intervention coverage, without taking into account subnational variation, particularly in multicountry assessments.^14 15^ This is partly because the cross-country comparability is more limited than for wealth, education or urban–rural residence, as the numbers and sizes of the subnational units may vary considerably within and between countries.

Advances in geospatial methods provide an opportunity to enhance the use of subnational analyses through thematic mapping, spatial analyses and modelling and eventually live systems.^16^ Mapping of coverage by administrative area has been common practice in many countries. A study of subnational DHS data from 27 countries in SSA showed the importance of contiguous geographical areas and cross-border associations for RMNCH indicators including immunisation coverage and care-seeking behaviours for child illness.^17^ Bayesian geospatial modelling with more than 25 covariates produced annual estimates of immunisation coverage in children 12–23 months for 5 by 5 km clusters in 52 countries in Africa during 2000–2016.^18^ These granular estimates were used to obtain coverage levels and trends for all districts, showing that, despite the overall progress, very few countries had reached Global Vaccine Action Plan target of over 80% in every district.^19^ At present, however, few countries make programmatic use of geospatial estimates for health indicators.

## Large subnational inequalities

To provide a general overview of current subnational inequalities in SSA, we computed the RMNCH composite coverage index (CCI), which has been used extensively in studies of inequalities in coverage and by the Countdown to 2030 for Women’s, Children’s and Adolescents’ Health. The CCI was designed to provide a general picture of inequalities in RMNCH coverage, summarising the programme performance for multiple intervention areas with a single measure, which is robust and strongly correlated with child mortality.^20 21^ The CCI is an average of four equally weighted intervention areas with eight maternal and child health interventions along the RMNCH continuum of care: family planning (demand satisfied for modern methods among currently married women), maternal and newborn care (four or more antenatal care visits, skilled birth attendance), immunisation (BCG, three doses of pentavalent and measles vaccinations) and treatment of sick children (oral rehydration solution for diarrhoea and care-seeking at a health facility for children with suspected pneumonia). Details of the CCI computation are shown in online supplementary appendix 1.

10.1136/bmjgh-2019-002232.supp1 Supplementary data

**Figure 1:**
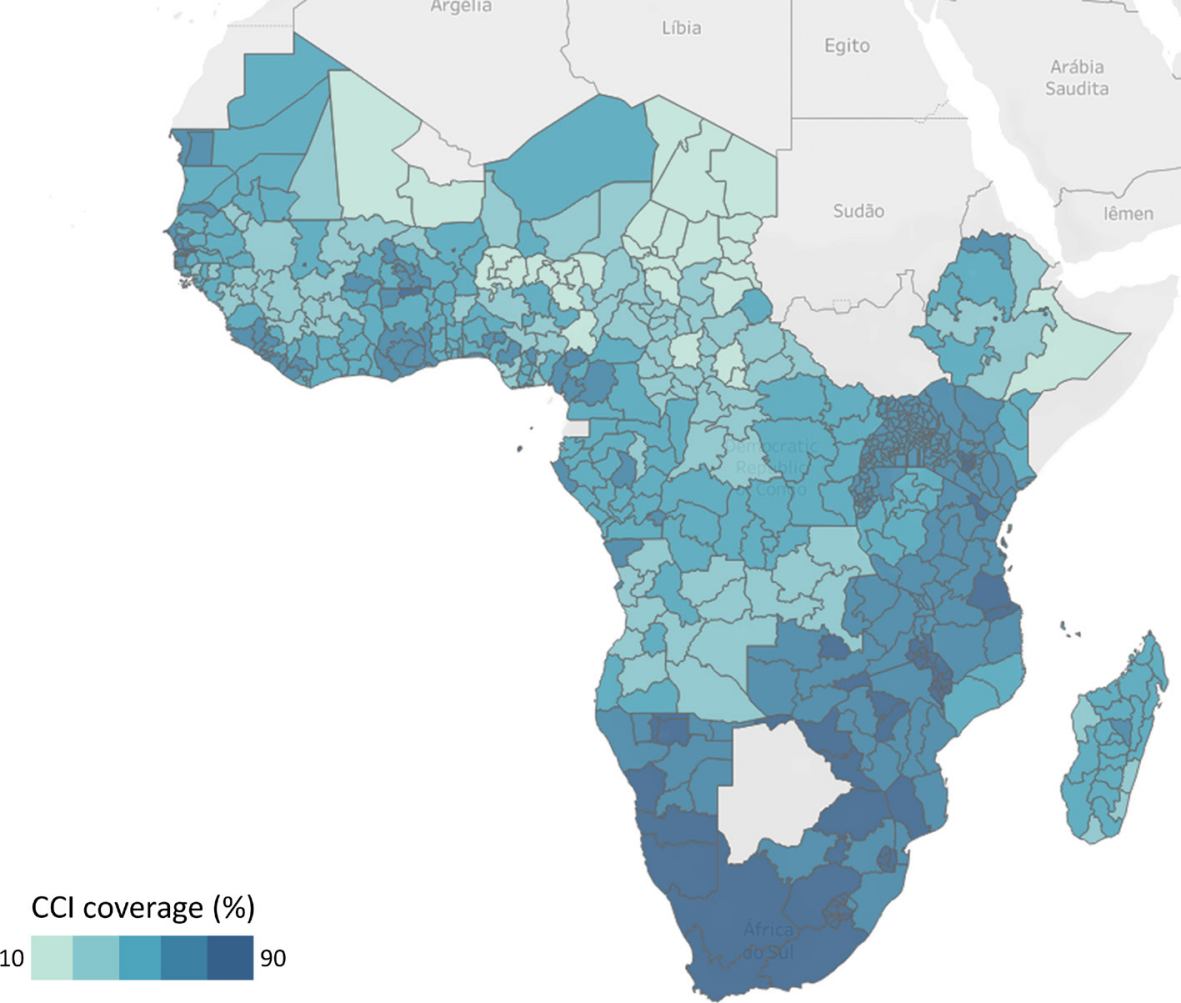
Map of composite coverage index (CCI) by subnational units in 39 countries, sub-Saharan Africa, most recent household survey.

The most recent national survey conducted in each of 39 countries in SSA was used to analyse the inequality gaps across subnational units. The survey years range from 2008 in Madagascar to 2017 in Senegal, with a median year in 2014. We grouped the countries into four geographical subregions based on the United Nation Population Division classification: Eastern, Southern, Central and West.^22^ Figure 1 maps the CCI by subnational unit based on the most recent surveys. Details of the country surveys, subnational units and CCI levels are shown in online supplementary appendix 2. The national CCI ranges from 28% in Chad to 83% in Eswatini. Countries in Southern Africa had the highest coverage, followed by Eastern Africa, West Africa and Central Africa.

**Figure 2:**
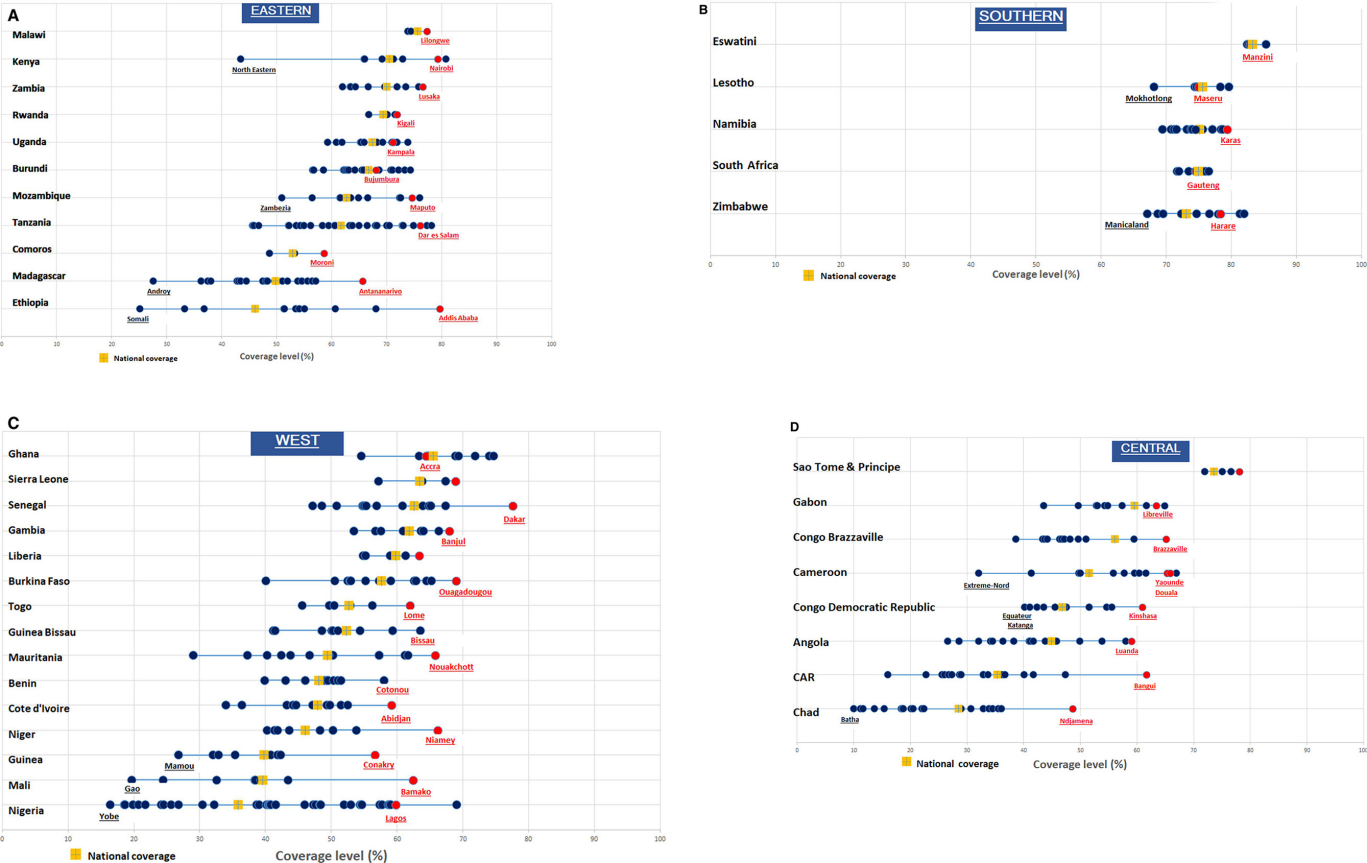
Reproductive, Maternal, Newborn and Child Health coverage (composite coverage index) by country and subnational unit in Eastern, Southern, West and Central Africa, most recent survey, capital regions highlighted in red. Some non-capital subnational units were highlighted to only flag some extreme values.

Figure 2A–D presents the CCI by subnational unit, ordered by national CCI within each SSA subregion. Each country line shows the CCI values for the subnational units and highlights the national CCI and the subnational area that includes the capital city. There is wide variation between countries, ranging from huge subnational disparities in countries such as Nigeria and Ethiopia to almost none in Malawi, Rwanda, Liberia and in all Southern Africa countries.

Because the number of subnational units varies greatly between countries (from 3 in Malawi to 37 in Nigeria), and the different sizes of the population within the subnational units, the patterns of inequality are not directly comparable between countries. Countries with more subnational units may have greater inequality irrespective of country surface area and population size (online supplementary appendix 6). The description of the gaps within countries does, however, provide a picture of the challenges that national programmes face in achieving universal coverage of essential RMNCH interventions.

## Capital city advantage

Regions containing the capital city are generally expected to have higher coverage rates than other subnational areas, even though the urban slums comprise a large and increasing proportion of the population with poor living conditions and health outcomes.^23 24^ The urban poor in the capital region should have improved access to essential RMNCH interventions, such as family planning, skilled birth attendance and immunisation, resulting in higher coverage. The coverage advantage of the capital region is clearly associated with the national level of CCI (figure 2A–D). In all but three countries with low or very low coverage (national coverage below 60%), the capital region had the highest CCI and often by some margin (table 1). The exceptions were Nigeria, Cameroon and Gabon. In the intermediate and high coverage countries (national coverage at least 60%), the capital regions were leading in only 7 of the 18 countries and generally only by a small margin.

**Table 1:** Subnational inequality measures and RANK of the capital region, grouped by RMNCH coverage level (CCI, most recent country DHS with survey year)

Country	Subregion	Survey	Year	Units	CCI	MDMW*	Inequality pattern†		Capital highest
Very low coverage (<45%)									
Chad	Central	DHS	2014	21	28.4	8.2	1.8		y
CAR	Central	MICS	2017	17	35.3	9.7	7.0	Top	y
Nigeria	West	MICS	2016	37	35.9	11.5	13.6	Top	
Mali	West	MICS	2015	8	39.6	5.1	2.9		y
Guinea	West	DHS	2013	8	39.7	6.4	4.0		y
Angola	Central	DHS	2016	18	44.9	9.8	−4.2		y
Low coverage (45%–59%)									
Ethiopia	Eastern	DHS	2016	11	46.0	9.1	12.7	Top	y
Niger	West	DHS	2012	8	46.1	4.6	14.2	Top	y
DR Congo	Central	DHS	2014	11	47.1	5.1	7.0	Top	y
Cote d’Ivoire	West	MICS	2016	11	47.9	4.9	−2.6		y
Benin	West	MICS	2014	8	48.1	3.7			y
Mauritania	West	MICS	2015	13	49.4	11.0	−4.1		y
Madagascar	Eastern	DHS	2008	22	49.8	7.3	−6.3	Bottom	y
Cameroon	Central	MICS	2014	12	51.5	12.2	−3.9		
Guinea Bissau	West	MICS	2014	9	52.3	8.4	−0.1		y
Togo	West	DHS	2013	6	52.7	5.3	2.2		y
Comoros	Eastern	DHS	2012	3	52.9	4.5	1.5		y
Congo	Central	MICS	2014	12	56.1	8.3	−8.5	Bottom	y
Burkina Faso	West	DHS	2010	13	57.6	6.5	−6.2	Bottom	y
Gabon	Central	MICS	2012	10	59.5	4.8	−10.6	Bottom	
Liberia	West	DHS	2013	5	59.8	3.6	−1.4		y
Intermediate coverage (60%–69%)									
Tanzania	Eastern	DHS	2016	30	61.7	9.0	0.3		
Gambia	West	DHS	2013	8	61.9	3.6	−2.3		y
Senegal	West	DHS	2017	14	62.6	8.1	−0.4		y
Mozambique	Eastern	DHS	2011	11	62.7	6.2	10.7	Top	
Sierra Leone	West	DHS	2013	4	63.4	4.8	−0.7		y
Ghana	West	DHS	2014	10	65.5	4.0	−1.7		
Burundi	Eastern	DHS	2016	18	66.7	4.6	−2.5		
Uganda	Eastern	DHS	2016	15	67.4	3.4	−1.8		
Rwanda	Eastern	DHS	2014	5	69.6	1.4	−0.6		y
High coverage (≥70%)									
Zambia	Eastern	DHS	2013	10	70.1	4.7	−1.6		y
Kenya	Eastern	DHS	2014	8	70.4	4.8	−16.6	Bottom	
Zimbabwe	Southern	DHS	2015	10	73.1	4.2	2.8		
Sao Tome and Principe	Central	MICS	2014	4	73.6	2.1	2.8		y
South Africa	Southern	DHS	2016	9	74.5	1.5	−0.7		
Namibia	Southern	DHS	2013	13	74.5	2.3	−0.3		
Lesotho	Southern	DHS	2014	10	75.2	1.6	−4.0		
Malawi	Eastern	DHS	2015	3	75.6	1.5	0.0		y
Eswatini	Southern	MICS	2014	4	83.3	0.5	1.4		

*See online supplementary appendix 3 for details on the MDWM.

†See online supplementary appendix 5 for details on the inequality pattern.

CCI, composite coverage index; DHS, Demographic and Health Survey; MDMW, weighted mean difference from overall mean; MICS, Multiple Indicator Cluster Survey; RMNCH, Reproductive, Maternal, Newborn and Child Health.

## Identifying programme-relevant patterns of inequality

Extensive research on the association between RMNCH coverage and household wealth has led to the identification of distinct patterns of inequalities by wealth quintiles.^25–27^ The patterns have major implications for policies and programmes. Top inequality (or mass deprivation) occurs when national coverage is low and the wealthiest have substantially higher coverage than the rest of the population. Interventions should be targeted to the whole population. Bottom inequality (or marginal exclusion) occurs when high coverage is reached but the poorest lags behind considerably. Interventions must be targeted to those that are left behind. A linear pattern is observed when coverage increases more regularly by wealth quintile. If the differences with the national coverage are large, programmes should focus on the whole population with further targeting of the poorest performing subnational units. If the variation is small, a whole population approach is more effective. Universal coverage is reached if coverage is high in all wealth quintiles. Absolute inequalities tend to be highest when national coverage is about 50%, as was shown for institutional deliveries.^26^ We adapted this approach to assess inequalities by subnational units in each country.

First, we selected a measure that describes the inequality of the whole distribution of subnational units. A wide range of measures is available to summarise distributions of subnational health inequalities.^28^ We used the population weighted mean difference from overall mean (MDMW), an absolute measure of inequality, which is the sum of absolute difference of each subnational unit’s CCI from the national CCI, multiplied by the proportion of the population in the subnational unit.^27 28^ We multiplied the MDMW values by 100 to facilitate interpretation. Detailed computation of the MDMW is provided in online supplementary appendix 3. We also computed the Theil index, which is a relative inequality measure.^28–30^ These results are presented in online supplementary appendix 4 and were quite similar to those from the MDMW analyses. Online supplementary appendixes 7 and 8 also show the association between the MDMW and the national CCI. As expected, higher levels of inequality occur at lower levels of national coverage.

Second, to capture the tails of the distribution of inequality, we used the inequality pattern index proposed by Victora *et al*^26^ which describes the difference between two gaps: between the top-performing subnational unit and the national coverage and between the bottom-performing subnational unit and the national coverage (online supplementary appendix 5). Positive values imply top inequality, while negative values are associated with bottom inequality. We used plus or minus five percentage points as an arbitrary cut-off point to identify top and bottom inequality.

Given the expected association between levels and patterns, we grouped the countries according to national coverage level (table 1). The MDMW shows the large inequalities between subnational regions in several countries. Most countries with high inequality are found in West and Central Africa, but with considerable variation between countries within the subregion. Cameroon, Nigeria and Mauritania have the most unequal subnational coverage, followed by Angola, Central African Republic and Ethiopia. The MDMW is negatively correlated with the national CCI (Pearson’s r=−0.69, online supplementary appendix 7). All countries in the high and intermediate coverage group had relatively small MDMW values (below 5), with the exceptions of Tanzania, Senegal and Mozambique.

Top inequality is observed in six countries including five with very low or low coverage such as Nigeria, Ethiopia and the Democratic Republic of Congo, the three largest population countries in SSA. Mozambique is the only country with a CCI greater than 60% that had top inequality (Maputo). In these countries, RMNCH programmes should prioritise increasing coverage in all subnational units, while targeting specific regions is less desirable.

Bottom inequality is most prominent in countries with a CCI near 50%. For instance, the coverage rates in the bottom regions in Burkina Faso, Congo and Gabon were about 25% below the best-performing region and more than 15% below the national CCI. Kenya has high coverage, but one province (North Eastern) is lagging behind tremendously with a gap of 27% with the national average.

Eswatini is the only country approaching universal coverage, characterised by a CCI over 80% in all subnational units. While 100% remains the target for the coverage of essential intervention, coverage from 80% may be considered as near universal because of measurement issues. Several coverage indicators rarely reach 100% because the determination of the population in need (denominator) is not perfect in the survey instrument, especially for coverage of family planning with modern methods and treatment-seeking behaviour for common childhood illnesses.^21^

## Limited reductions in subnational inequality

Research studies of long-term trends in intervention coverage in specific countries have shown overall improvements during 1990–2010, but also noted that subnational gaps tended to persist.^11 31^ We used data from 34 countries with two surveys since 2000 to ascertain trends in CCI inequality. In case of more than two surveys since 2000, we selected the surveys conducted about 10 years before the last survey. The median year of the first survey was 2006, and 2014 for the last.

To assess the trends in subnational inequalities by country, we plotted the average annual absolute rate of change in coverage against the average annual absolute change rate in subnational inequality (online supplementary appendix 3). Overall, the CCI increased from 50% to 59% between the first and second survey, improving at an annual rate of change of 2.0%, but the inequality, as measured by the MDMW, only improved slightly during 2004–2015.

**Figure 3:**
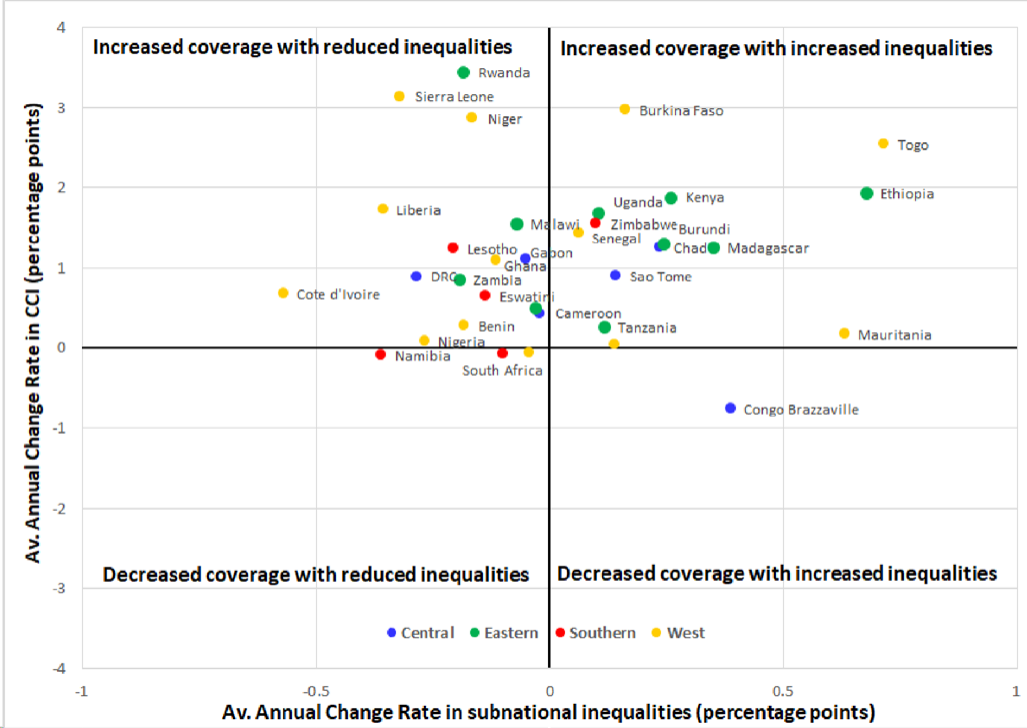
Change in subnational inequality, as measured by the average annual change rates in overall coverage and in subnational inequalities, 34 countries in sub-Saharan Africa. CCI, composite coverage index.

Figure 3 is a four-quadrant graph indicating country average annual performances in both coverage and subnational inequalities. In 16 countries, coverage increased and subnational inequality reduced. In 14 countries, there was an increase in coverage with increased subnational inequality. The third group includes Namibia, South Africa and Mali where coverage has decreased with reduced subnational inequality. Only in Congo Brazzaville, coverage decreased over time with increased subnational inequality.

Many countries with large inequalities (table 1) are fragile states or countries that have gone through a recent conflict or still have an ongoing conflict. Previous analyses of the Countdown have shown the impact of conflicts on the CCI with possible acceleration of improvements in the post-conflict phase.^32^ Inequalities in the CCI also tend to be greater in conflict-affected countries.^33^ In other Countdown analyses, it was shown that levels and wealth-related equity in RMNCH coverage were positively associated with better governance, especially political stability and absence of violence, and with level of economic development of the country, and inversely associated with country surface area.^34^ We also considered the Kaufman governance index which consists of six composite indicators of broad dimensions of governance, such as accountability, political stability, government effectiveness and control of corruption.^35^ In our analysis with MDMW as the dependent variable, controlling for the number of subnational units in a country, we found a moderately strong association between governance and subnational inequalities (online supplementary appendix 6). Countries with better governance had lower subnational inequalities in RMNCH coverage, which could be interpreted as evidence of greater government efforts to reach all women and children with essential services.

We also examined the association between income inequality, as measured by the Gini index, and the CCI (online supplementary appendix 6).^36^ There was no significant association between the Gini coefficient and subnational inequalities. Several countries in southern Africa had the highest income inequalities but lowest levels of subnational inequalities. Some countries with relatively small Gini coefficient such as Ethiopia, Tanzania and Mauritania had high levels of RMCNH coverage. It appears that the coverage of essential RMNCH interventions is protected from adverse consequences of income inequality on health equity which may be associated with the additional investments in RMNCH interventions during the MDGs era. The observation that RMNCH inequalities were not associated with income inequality is encouraging, perhaps showing how high RMNCH coverage can be reached among disadvantaged populations in adverse circumstances. Other approaches, such as estimated absolute income from surveys, may throw further light on the complex association between income and RMNCH coverage inequalities.^37^ A comprehensive effort to explain subnational disparities requires more research that includes economic and governance and also a wide range of explanatory variables such as disease epidemiology, country geography and climate, and sociocultural heterogeneity.

## Conclusion

Subnational inequality in the coverage of RMNCH interventions is an important public health issue in many countries. The inequalities between regions or provinces varied greatly between countries. Disparities were smallest in high coverage countries in Southern Africa, Rwanda and Malawi and in several small population countries such as the Benin, Gambia and Liberia. Many countries however had large inequalities between regions and provinces, some with clear patterns of bottom inequality in remote regions or top inequality in especially capital cities in low coverage countries. It is important to take into account that subnational inequalities are not directly comparable between countries, as they are associated with the number of subnational units and population size, as shown in our analyses. We used summary measures of inequality that take into account population size of the respective units, but the number of units still has a major effect.

There was a moderately positive trend towards reductions in subnational inequality during 2006–2014, with 16 of the 34 countries reducing inequalities. Countries with low coverage and high levels of inequality in the initial survey still had high inequality in the most recent survey despite improvements in coverage. Many of those countries were fragile states and affected by conflict, showing that major efforts are needed to reach everyone with high coverage of RMNCH interventions.

The analysis also shows the challenges ahead in efforts to reach universal coverage of essential interventions. As countries move towards reaching all individuals, the analysis of coverage levels and inequalities by administrative units such as region or province will not be sufficient to detect progress and guide targeted efforts. Countries need information from smaller administrative units, such as districts, to monitor progress towards universal coverage. Unless household surveys are very large, it will not be possible to obtain reliable estimates of interventions coverage at district level. Coverage estimates based on health facility data may be an alternative option, but to-date, data quality as well as accurate estimation of target population are an obstacle in many countries.^8^

In addition, we did not take into account sampling error in presenting the coverage index for subnational levels. We opted to assess the average annual rates of change for a large number of countries without sampling errors to assess the overall direction and speed of change, as has been done in other studies,^38^ resulting in a sufficiently clear picture of lack of major change in SSA as a whole. The CCI itself is a proven measure of the coverage of RMNCH interventions, but it has to be noted that the specific interventions may show different inequalities, and the quality of care is not taken into account for most indicators. There may be further inequalities in the quality of care between the subnational units.

In summary, high national coverage levels can only be reached by closing the gap between high coverage regions, such as the capital cities, and all other regions, as has happened in several countries. The persistence of the country patterns of subnational inequality over time, however, does suggest that this will require a major effort by country programmes.
