# Supplementary material for: Large and persistent subnational inequalities in reproductive, maternal, newborn and child health intervention coverage in sub-Saharan Africa
Source: BMJ Glob Health. 2020 Jan 26;5(1):e002232. doi: 10.1136/bmjgh-2019-002232 (PMC7042572; doi:10.1136/bmjgh-2019-002232)

Appendix 1: Details of the Coverage Composite Index (CCI) computation

The CCI is the weighted average of the percentage coverage of eight interventions along four stages of the continuum of care: reproductive care; maternal care; childhood immunization; and management of childhood illness. The interventions are: (i) modern family planning coverage (DFPSmo); (ii) at least four antenatal care visits by a skilled provider (ANC4); (iii) skilled birth attendant (SBA); (iv) bacille Calmette–Guérin (BCG) vaccination; (v) MSL stands for measles immunization; (vi) DPT3 is three doses for diphtheria-tetanus-pertussis immunization; (vii) ORS means oral rehydration salts for diarrhea; and (viii) CAREP is the care-seeking for suspected acute respiratory infection. The CCI is calculated as below:

$$CCI = \frac{DFPSmo + \frac{ANC4 + SBA}{2} + \frac{BCG + MSL + 2 * DPT3}{4} + \frac{ORS + CAREP}{2}}{4}$$

See the references cited

- Boerma T, AbouZahr C, Evans D, et al. Monitoring Intervention Coverage in the Context of Universal Health Coverage. PLOS Medicine 2014;11(9):e1001728. doi: 10.1371/journal.pmed.1001728
- Wehrmeister FC, Restrepo-Mendez M-C, Franca GV, Victora CG, Barros AJ. Summary indices for monitoring universal coverage in maternal and child health care. Bulletin of the World Health Organization. 2016;94(12):903.
- Barros AJD, Victora CG. Measuring Coverage in MNCH: Determining and Interpreting Inequalities in Coverage of Maternal, Newborn, and Child Health Interventions. PLOS Medicine. 2013;10(5):e1001390.

Appendix 2. Details of the country surveys, subnational units and CCI levels

Table 1. Surveys included and number of subnational by country and African region

African region	Country	Surveys included	Number of subnational units	Coverage level (%) (recent survey)
Central	Angola	DHS 2015	18	44.9
Central	Cameroon	DHS 2004-MICS 2014	12	51.5
Central	Central African Republic	MICS 2010	17	35.3
Central	Chad	DHS 2004-DHS 2014	21	28.4
Central	Congo Brazzaville	DHS 2011-MICS 2014	12	56.1
Central	Congo Democratic Republic	DHS 2007-DHS 2013	11	47.1
Central	Gabon	DHS 2000-DHS 2012	10	59.5
Central	Sao Tome and Principe	DHS 2008-MICS 2014	4	73.6
Eastern	Burundi	DHS 2010-DHS 2016	18	66.7
Eastern	Comoros	DHS 2012	3	52.9
Eastern	Ethiopia	DHS 2005-DHS 2016	11	46.0
Eastern	Kenya	DHS 2008-DHS 2014	8	70.4
Eastern	Madagascar	DHS 2003-DHS 2008	22	49.8
Eastern	Malawi	DHS 2004-DHS 2015	3	75.6
Eastern	Mozambique	DHS 2003-DHS 2015	11	62.7
Eastern	Rwanda	DHS 2005-DHS 2014	5	69.6
Eastern	Tanzania	DHS 2004-DHS 2015	30	61.7
Eastern	Uganda	DHS 2006-DHS 2016	15	67.4
Eastern	Zambia	DHS 2001-DHS 2013	10	70.1
Southern	Eswatini	DHS 2006-MICS 2014	4	83.3

Southern	Lesotho	DHS 2004-DHS 2014	10	75.2
Southern	Namibia	DHS 2006-DHS 2013	13	74.5
Southern	South Africa	DHS 1998-DHS 2016	9	74.5
Southern	Zimbabwe	DHS 2005-DHS 2015	10	73.1
West	Benin	DHS 2006-MICS 2014	12	48.1
West	Burkina Faso	DHS 2003-DHS 2010	13	57.6
West	Cote d'Ivoire	DHS 2011-MICS 2016	11	47.9
West	Gambia	DHS 2013	8	61.9
West	Ghana	DHS 2003-DHS 2014	10	65.5
West	Guinea	DHS 2005-DHS 2012	8	39.7
West	Guinea Bissau	MICS 2014	9	52.3
West	Liberia	DHS 2007-DHS 2013	5	59.8
West	Mali	MICS 2009-MICS 2015	8	39.6
West	Mauritania	MICS 2011-MICS 2015	13	49.4
West	Niger	DHS 2006-DHS 2012	8	46.1
West	Nigeria	MICS 2007-MICS 2016	37	35.9
West	Senegal	DHS 2005-DHS 2017	14	62.6
West	Sierra Leone	DHS 2008-DHS 2013	4	63.4
West	Togo	MICS 2010-DHS 2013	6	52.7

Appendix 3. Computation of the weighted mean difference from overall mean (MDMW), Average annual absolute rate of change in coverage (AARC) and Average annual absolute rate of change in subnational inequality (AARM)

- The MDMW is computed as follows: $\mathrm{MDMW} = \sum_{\mathrm{i}}^{\mathrm{n}} p_i \ |\mathrm{CCI}_i - \mathrm{CCI}| * 100$, where, for subnational unit i, p_i is the proportion of the population and CCI_i is the coverage level.
- The average annual absolute rate of change in coverage is given by: AARC = 100*(CCI_2-CCI_1)/ (Y_2-Y_1), where CCI_2 is the coverage level in Year 2 (Y_2) and CCI_1, the coverage level in Year 1(Y_1).
- The average annual absolute change rate in subnational inequality is AARM = 100*($MDMW_2$-$MDMW_1$)/ (Y_2-Y_1) where $MDMW_2$ is the level of subnational inequality in Year 2 (Y_2) and $MDMW_2$, the level of subnational inequality in Year 1(Y_1).

Appendix 4. Theil Index analyses

The Theil Index (TI) is an inequality measure that takes into account the proportion of the population in each subnational unit and the ratio of the health indicator prevalence (CCI in this study) in each unit to the national mean health indicator prevalence. The TI has a minimum value of 0 (no subnational inequality); the value becomes larger as the subnational inequality increases. In this study, the TI values have been uniformly multiplied by 1000 to facilitate interpretation. The TI is computed as below:

$$\mathrm{TI} = 1000\ \mathrm{X} \sum_{\mathrm{i}}^{\mathrm{n}} p_i\ \mathrm{r}_i\ \ln\ (\mathrm{r}_i)$$

where, for subnational unit i, p_i is the proportion of the population, and r_i is the ratio of the CCI in subnational unit i to the overall CCI at country level.

Looking at the Theil Index, results presented in Figure A1 shows that the largest subnational inequalities were observed in West and Central Africa regions, with Nigeria, Chad and Central African Republic having the largest gaps. Countries in Southern Africa seem to have minimal subnational health inequalities, followed by countries in Eastern Africa.

Specifically, Nigeria remained the country with the largest subnational inequality in West Africa while Liberia presented the smallest inequality in the region. In Central Africa, Chad and CAR exhibited the largest subnational inequality, compared to Sao Tome & Principe where the TI was smallest. Looking at Eastern Africa, the largest subnational inequality was noted in Ethiopia while Malawi had the smallest TI value. In Southern Africa, subnational inequalities were lowest in Eswatini and largest in Zimbabwe.

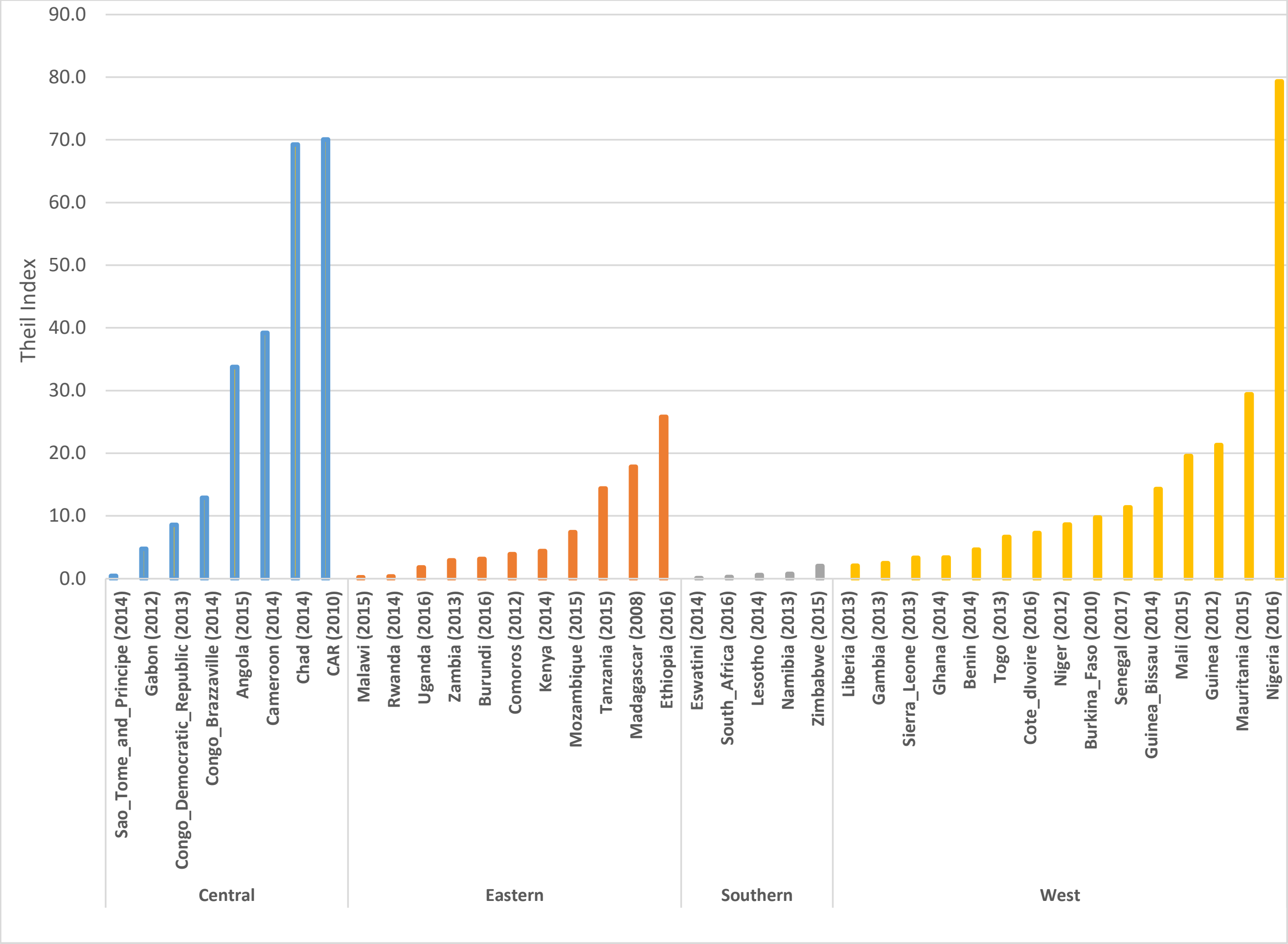

Figure A1. Subnational inequality for the RMNCH composite coverage index: Theil Index (TI) in 39 countries, sub-Saharan Africa, most recent national survey, with countries grouped by subregion.

Appendix 5. The Inequality pattern index

The inequality pattern index was proposed by Victora et al. (Victora CG, Joseph G, Silva IC, et al. The inverse equity hypothesis: analyses of institutional deliveries in 286 national surveys. *American journal of public health* 2018;108(4):464-71.). It describes the difference between two gaps: between the top performing subnational unit and the national coverage and between the bottom performing subnational unit and the national coverage

Index = $(C_{top} - C_n) - (C_n - C_{bottom}) = C_{top} + C_{bottom} - 2C_n$, where C_n is national coverage, and C_{top} and C_{bottom} represent respectively the highest and lowest subnational coverage.

Appendix 6. Co-variates of inequality

The first linear regression analysis was conducted to assess the association of the number of subnational units, country size (log), country population size (log) and the proportion of urban population with the mean distance from the mean, weighted (MDMW). The table below shows the results from the bivariate analysis and the multi-variate analysis including all three co-variates to predict MDMW. The results show that the main factor affecting the size of MDMW is the number of subnational units: the more units, the greater the inequality.

	Bivariate			Multi-variate		
**Covariates**	**Coef.**	**(95% CI)**	**p-value**	**Coef.**	**(95% CI)**	**p-value**
Number of subnational units	0.27	(0.15;0.38)	<0.001	0.23	(0.10;0.36)	0.001
Country size (log)	0.77	(0.28;1.25)	0.003	0.49	(-0.19;1.18)	0.152
Population (log)	0.72	(0.06;1.38)	0.034	-0.25	(-1.17;0.68)	0.594
Urban population	0.03	(-0.03;0.08)	0.385	0.02	(-0.03;0.07)	0.392

In the subsequent analysis of association of other factors with the subnational inequality as measured by the MDMW we controlled for the number of subnational units. The two tables below show the results of the linear regression analysis to assess the association with the income inequality (GINI coefficient) and the Kaufmann index of governance, both bivariate and after controlling for the number of subnational units. Only the Kaufmann index shows a statistically significant association.

	Bivariate			Multi-variate		
**Covariates**	**Coef.**	**(95% CI)**	**p-value**	**Coef.**	**(95% CI)**	**p-value**
GINI coefficient	-0.07	(-0.20; 0.06)	0.28	-0.07	(-0.17;0.03)	0.150
Number of subnational units				0.27	(0.16;0.38)	<0.001

	Bivariate			Multi-variate		
**Covariates**	**Coef.**	**(95% CI)**	**p-value**	**Coef.**	**(95% CI)**	**p-value**
Kaufmann Control of Corruption index	-3.00	(-4.66;-1.33)	0.001	-2.34	(-3.72;-0.96)	0.001
Number of subnational units				0.23	(0.13;0.33)	<0.001

Appendix 7. Scatter plot of the inequality, measured with the Mean deviation from the mean (MDMW) by the RMNCH Composite Coverage index (CCI), with linear and exponential regression lines.

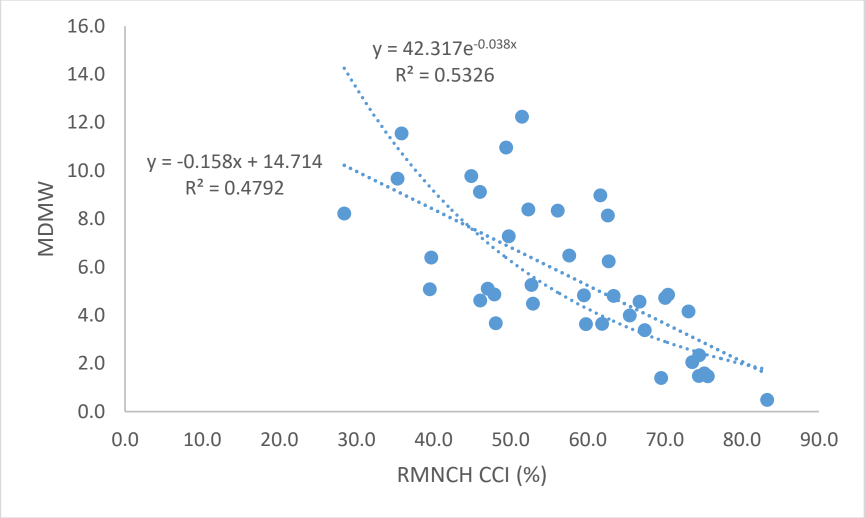

Appendix 8. Scatter plot of the correlation between MDMW and national CCI with linear regression line

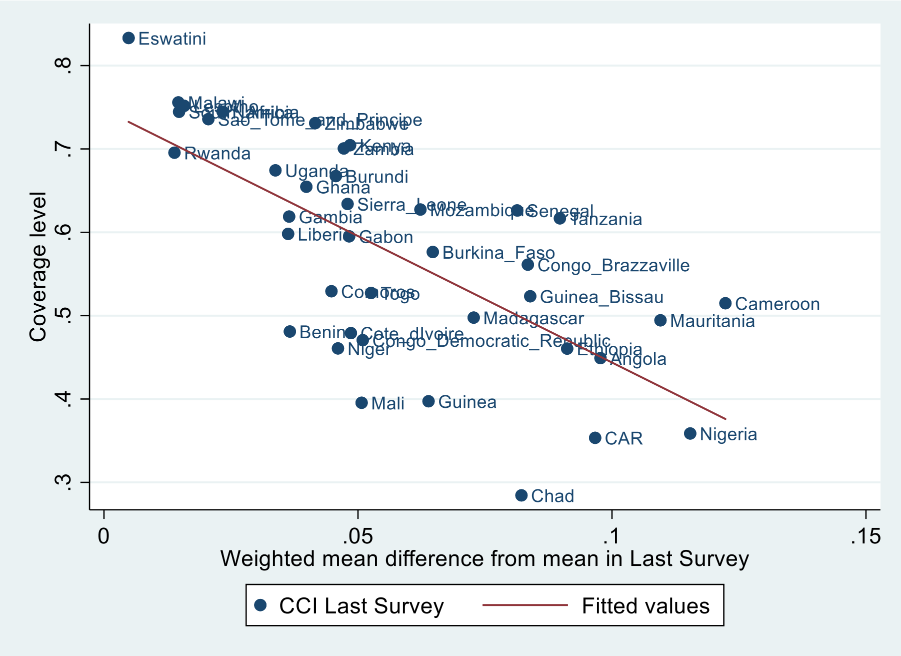

Supplement: Supplementary data [file bmjgh-2019-002232supp001.pdf]
